# Analyzing gendered patterns in sentiments in comments under STEM YouTube channels

**DOI:** 10.3389/frai.2026.1790240

**Published:** 2026-05-07

**Authors:** Isha Karn, N. Ilakiyaselvan, V. Kalyanasundaram, A. J. Keerthi

**Affiliations:** School of Computer Science and Engineering, Vellore Institute of Technology, Chennai, Tamilnadu, India

**Keywords:** gender representation, Latent Dirichlet Allocation, natural language processing, RoBERTa, sentiment analysis, STEM, YouTube comments

## Abstract

This study explores gender-based disparities in sentiments and discourse in comments on YouTube channels focused on science, technology, engineering, and mathematics (STEM). Although there have been considerable efforts toward achieving gender equality, biases and inequalities still persist in the representation of women in STEM, including in online communication spaces. A mixed-methods approach was used, based on a dataset of comments collected from over 100 curated STEM YouTube channels using the YouTube Data API. The comments were cleaned and preprocessed, and information regarding the gender of content creators was identified. The RoBERTa model was used for sentiment annotation, while Latent Dirichlet Allocation (LDA) was applied for topic modelling. The results show a clear difference in sentiment distribution between genders. Negative comments were more frequent in female-hosted channels and were often related to personal criticism, objectification, and appearance-based remarks, whereas comments on male-hosted channels were more neutral and focused on content. The findings indicate the presence of gender bias in online discussions related to STEM.

## 1 Introduction

Despite growing advocacy and policy initiatives for greater gender equity in STEM, women continue to be underrepresented in a variety of scientific and technological fields (Knobloch-Westerwick et al., 2013; Luntz, 2011; Zakaib, 2011; Schmuck, 2017). Previous studies have shown that structural issues, implicit bias, and stereotypical media depictions have continued to influence public views of women in science (Luntz, 2011; Schmuck, 2017). Although institutional changes have improved representation and accessibility, inequities continue not only in the workplace but also in science communication environments.

The development of digital media has revolutionized science communication, shifting the discourse from traditional media gatekeeping to collaborative online forums (Claussen et al., 2013; Lo et al., 2010; Nisbet and Scheufele, 2009). Digital media platforms such as YouTube allow STEM professionals and science communicators to connect with large audiences. However, the comment sections of such platforms are public forums where views, biases, and judgments are openly articulated. The analysis of audience sentiment across gendered hosts in these online forums is essential for investigating current trends of representation and bias in STEM communication.

Although there have been studies on the patterns of sentiment expressed on social media and the gender gap in STEM representation, there is a lack of large-scale research on the discourse of the audience under STEM-themed YouTube channels when comparing the two genders. Most studies are either based on the performance of sentiment classification or the feminist hashtag movements, with relatively fewer studies that combine sentiment analysis and thematic modeling.

The goal of this study is to analyse the responses to STEM channel videos in YouTube comments in order to look for patterns of sentiment, biases, stereotypes, and public perceptions of the hosts - of presenting as male and female. Understanding the nuanced interplay of sentiments toward hosts of different genders is the first step toward improving and fostering a more inclusive and equitable online environment and promoting diversity in STEM fields.

After exploring the research in the field, it is found that there is a significant lack of availability of a dataset that fosters holistic exploration of this domain. Curating a dataset from scratch from a platform as relevant as YouTube is bound to improve visibility and encourage further exploration. Furthermore, by employing an interdisciplinary approach involving both Statistical and Machine Learning methods along with Sociological Research, we aim to provide a novel methodology and act as ground for future research.

The data comprises of comments collected from a curated list of over a hundred influential YouTube channels spanning the Tech and Science categories. Utilizing the “Most Recent” videos ensure recency and captures a diverse range of current perspectives and discussions within the community. This approach toward data collection aims to provide a rich, balanced, and varied dataset for thorough analysis. Sentiment annotation classifies comments into Positive, Negative and Neutral sentiments using RoBERTa (Robustly Optimized BERT Approach) (Liu et al., 2019), a transformer approach that is suited for tasks concerning annotations. Additionally, themes, patterns and language use related to gender, biases, and stereotypes within comments are explored by conducting a fine-grained topic analysis using LDA (Latent Dirichlet Allocation) (Blei et al., 2003). The discussions will address ladies in STEM by outlining specific challenges, barriers, and opportunities for women in STEM based on the findings. This approach allows the proposed method of inquiry to reveal subtle insights underlying a complicated set of factors through the dynamics of digital engagement on YouTube.

Through these analyses, the research aims to draw actionable insights, implications, and recommendations to inform educational initiatives, policy interventions, and community engagement efforts aimed at promoting gender inclusivity, diversity, and representation in STEM fields. Identifying opportunities for future research, collaboration, and action will further contribute to addressing gaps, challenges, and emerging issues related to gender dynamics in digital discourse and STEM communication on YouTube.

## 2 Related works

Understanding the experiences and challenges faced by women in STEM education is an important precursor to exploring the effects of social media and scientific communication. Studies on sentiment analysis predominantly focus on improving detection techniques rather than delving into the analysis of the sentiments themselves. This emphasis on methodological advancement highlights the ongoing efforts to enhance the accuracy and efficiency of sentiment analysis tools. However, there is a noticeable gap in research, particularly concerning scientific communication and gender minorities in STEM.

Furthermore, previous research persists with a gender binary lens and focuses only on male or female content creators. The gender binary lens elides gender minority experiences or perspectives, causing researchers to have a warped perspective of sentiment dynamics in online engagement. Because of limited classifications as well as no options to identify as trans, or gender queer, the gender binary has skewed the available data on other gender identities. Too many studies published on platforms like Twitter have a problematic impact on sentiment analysis with oversaturation of research in areas on mainstream platforms, which positions gender as a binary and ignores the experiences of marginalized communities.

For example, recent changes to Twitter’s terms and the terms of how their API (Application Programming Interface) can be used have complicated sentiment analysis research. They have forced restrictions on data collected through their API, changed how data could be collected, and could risk the reliability and validity of findings on studies that have used Twitter.

### 2.1 Women and STEM education

Existing research indicates that societal stereotypes and biases contribute heavily to the underrepresentation of women in STEM fields. Gender stereotypes influence students’ self-perceptions and career aspirations. Griffith (2010) highlighted persistence rates as a crucial metric to discern retention for women and minorities in STEM majors compared to their male counterparts. They employed statistics and regression analysis and uncovered that a lower percentage of said women and minorities persisted in their majors. Regression analysis suggested that disparities in educational experiences played a significant role in these persistence rate discrepancies. Their findings also emphasized the importance of categorizing women across various STEM professions and considering their backgrounds.

Price (2010) focused on the instructor’s race and gender. They discovered that a Black instructor instructed the Black students. Interestingly, female students were less likely to persist when more of their STEM courses were taught by female instructors. However, Carrell et al. (2010) found that this negative correlation between female instructors and female student persistence did not hold true for high-ability students.

Evidence from Hoffmann and Oreopoulos (2009) suggested that female students were more sensitive to grades. They were less likely than male students to pursue additional STEM courses when their grades were lower. Students that took Advanced Placement (AP) classes in STEM fields in high school showed enhanced persistence while pursuing a STEM degree. However, college grades themselves did not appear to be strong predictors of persistence (Ost, 2010).

### 2.2 Discourse on female centric topics on social media

The landscape of sociological analysis within digital discourse surrounding women in STEM and related feminist movements is rich and multifaceted. Studies have shed light on various aspects of online interaction and gender dynamics. Amarasekara and Grant (2019) investigated the sentiment of YouTube comments directed toward female science communicators on STEM channels. Their research exposed a concerning gender imbalance. The study identified a significant underrepresentation of female voices. Out of 391 channels analyzed, only 32 were hosted by females. Interestingly, despite their limited presence, female science communicators received a disproportionately high number of comments per view compared to their male counterparts. A substantial portion of these comments were negative in nature, including confrontational, sexualized, focused on appearance, or critical of the female communicators.

Fouad and Alkooheji (2023) explored the potential of Twitter sentiment analysis to promote gender parity in STEM fields. Their work investigated how online interactions influenced women’s interest in STEM careers. They employed a self-curated dataset with hashtags as queries to gather relevant tweets. Like our approach, they used an annotation scheme involving RoBERTa. The analysis revealed that the collected tweets about women in STEM were mostly positive, suggesting a supportive online environment. Interestingly, the sentiment analysis showed a rise in positive comments during months celebrating women’s achievements, such as International Women’s Day.

Kaur and Sharma (2020) performed a sentiment analysis on English-language tweets using the hashtags #MeToo and #Women to compare the usage of hashtags and the distribution of sentiment. The authors used TextBlob to label the sentiment of the tweets as positive, negative, or neutral based on their polarity and then trained supervised machine learning classifiers such as Naïve Bayes, Support Vector Machine (SVM), Random Forest, and Logistic Regression. The authors reported that the best accuracy (92.42% for #MeToo and 95.62% for #Women) was obtained using SVM, mainly focusing on the comparison of the performance of the classifiers. The authors’ study mainly concentrated on the overall polarity of the sentiment and the popularity of the hashtags, without delving into the thematic discourse patterns, bias, or gendered interpretive dynamics of the comments. The current study, on the other hand, goes beyond the comparison of classifier performance and incorporates state-of-the-art transformer models for sentiment analysis and topic analysis to explore the gendered discourse patterns in STEM-related online communities.

Rodriguez-Sanchez et al. (2020) investigated misogynistic language within Spanish tweets. They leveraged a Bi-LSTM (Long Stort-Term Memory) network for sentiment analysis, with the goal of detecting hate speech and various forms of misogyny. They self-curated a Spanish tweet dataset related to the #MeToo movement. This focused approach allowed them to train their models on tweets containing sexism or misogynistic language. Bi-LSTM models are adept at understanding the sequential nature of language. The study compared their Bi-LSTM model with a pre-trained model called mBERT. mBERT achieved the best accuracy (0.74%) for detecting misogyny within the dataset. The model struggled with tweets that used irony or contained words with ambiguous meanings. Lack of context beyond the single tweet and the small dataset size also impacted the accuracy of misogyny detection.

### 2.3 Methods for analyzing sentiments

Researchers reviewed different sentiment analysis techniques and methods across various application areas and tasks (Cui et al., 2023). They explored methods based on lexicons, rules, parts of speech, term position, statistical techniques, supervised and unsupervised machine learning methods, along with deep learning methods like LSTM (Long Short-Term Memory), CNN (Convolutional Neural Networks), RNN (Recurrent Neural Networks), DNN (Deep Neural Networks), BERT (Bidirectional Encoder Representions from Transformers), and other hybrid approaches.

Turney and Littman (2003) introduced a lexicon-based algorithm called ISA (semantic polarity) that relied on a predefined dictionary of words and their sentiment associations to determine the overall sentiment of a piece of text. This method reached an accuracy of 82.80%. In an analogous way, Yang et al. (2013) offered a model called SOPMI that also leveraged a lexicon-based approach and reached an accuracy of 92.40% on Chinese hotel reviews dataset. These lexicon-based methods offered a reasonable starting point due to their ease of use. However, a key limitation was their heavy dependence on the quality and comprehensiveness of the chosen dictionary. Furthermore, the algorithms assign sentiment scores to words independently, ignoring the order or context in which they appeared in the sentence.

CNN (static and multichannel model) (Kim, 2014), VDCNN (Conneau et al., 2017) and Seq-CNN (Johnson and Zhang, 2015) demonstrated significant performance in sentiment analysis tasks. VDCNN and Seq-CNN both achieved accuracies of 95.72 and 91.925%, respectively, on Yelp and the IMDB dataset. CNNs effectively processed data with speed and captured local patterns and relationships between words within a sentence. This allowed them to automatically extract sentiment-bearing features from text data, making them suitable for short-form text. Incorporating additional task-specific features could further enhance their performance.

RNNs offered a significant advantage over CNNs for sentiment analysis. LSTM and oh-2LSTMp models (Tang et al., 2015; Day and Lin, 2017; Johnson and Zhang, 2016) achieved accuracies of 94.00 and 94.06% on Google Play Customer Reviews and IMDB datasets, respectively. Unlike CNNs, RNNs processed text data word by word, effectively integrating information from adjacent positions. This allowed for considering the context of a word in relation to surrounding words, leading to a more nuanced understanding of sentiment, especially for longer sentences where word order plays a crucial role. However, RNNs might not effectively distinguish the importance of different word cues within a sentence. Some words might hold more weight in conveying sentiment than others, and an ideal model would be able to prioritize these cues.

Hybrid neural network models aimed to combine the strengths of different architectures like CNNs and RNNs (or even feature-based approaches like SVMs). The CNN-SVM model achieved 65.96% accuracy on online reviews for aspect-based sentiment analysis in Hindi (Akhtar et al., 2016). HN-ATT with GRU reached 71.00% accuracy on a Yelp dataset (Yang et al., 2016). Here, the GRU unit (a type of RNN) captured contextual information, while the attention mechanism (ATT) might have focused on crucial sentiment-bearing words. Despite their potential, hybrid models have not always achieved significant breakthroughs compared to simpler models. This could be due to the scarcity of large datasets annotated for sentiment analysis, especially for specific tasks or languages. Training complex hybrid models often requires vast amounts of labeled data to function optimally category sentiment analysis produce their highest f1 score of 80.00% on the public dataset for aspect-category sentiment analysis from AI challengers (Liao et al., 2021). FT-RoBERTa is also a more sentiment-word-oriented model and would benefit Aspect Based Sentiment Analysis tasks (Dai et al., 2021). It achieves significant accuracy in 4 different languages and achieves their highest accuracy of 87.52%. The advantages of these models occur because of their strength with pre-trained language representation as deep bidirectional Transformers, which can leverage both text and aspect tokens to capture not only the meanings of the words but also the relationship among the words and their meanings according to aspect or categories that can be used to enhance accuracy in sentiment analysis tasks. However, in addition to their advantages, there are limitations associated with RoBERTa-based sentiment analysis models such as computational complexity and resource-intensive nature of deep learning models that may influence training time, inference time, and number of resources required for the training, and other tasks. Moreover, not all these models are equally effective for every task, and even across datasets; making it essential to evaluate models and tune them accordingly.

## 3 Methods

**Figure 1:**
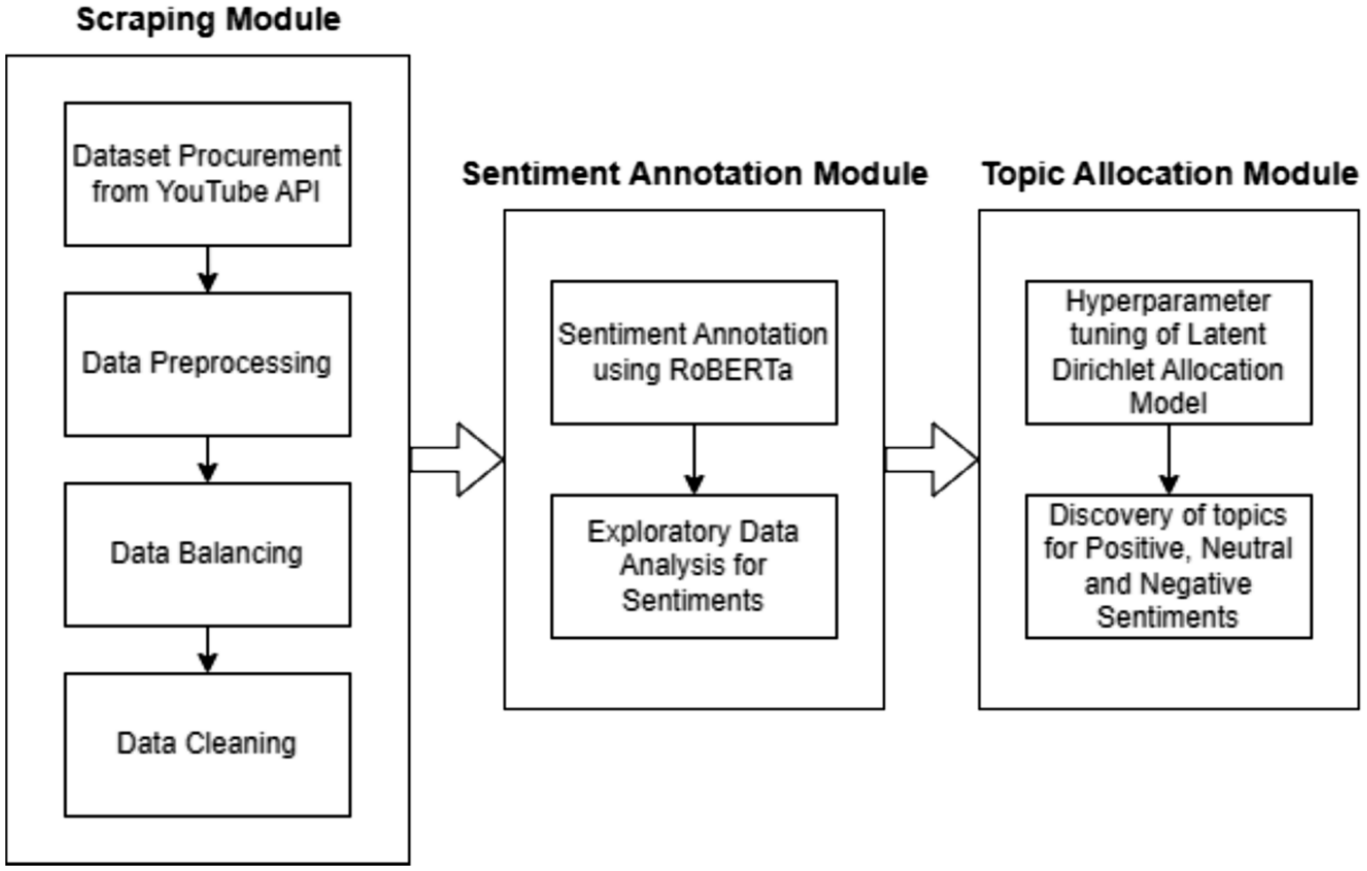
Schematic for the methodology followed.

This study’s methodology uses a multistep process of collected, processed, and analyzed data to get deeper insights into the sentiment dynamics and topic distributions in a STEM greater YouTube channel context. A schematic diagram is utilized to illustrate the sequential flow of the proposed methodology, depicting key steps such as data procurement, pre-processing, sentiment analysis, and topic modeling (Figure 1).

### 3.1 Dataset procurement

A comprehensive approach was taken to procure data from a diverse range of YouTube channels, encompassing a total of 131 channels with varying subscriber counts. Channels were selected through meticulous manual curation, drawing insights from blog posts, articles, and other reliable sources (Sung, 2021; Shenova Fashion, 2026; Kaye, 2017; Social Blade, 2026). The primary criterion for selection was the relevance of the channels to the Science and Technology category, with rankings sourced from Social Blade (2026). This process resulted in the generation of a top 100 list of channels, ensuring representation from a broad spectrum of content creators.

To ensure the dataset’s diversity and inclusivity, smaller channels with subscriber counts ranging from 0.0 to 0.5 million were also included in the list. This decision aimed to prevent the neglect of emerging channels and provide a balanced representation across the subscriber spectrum. Videos were limited to the top 10 most recent uploads for each channel. The scraping process took place on 11/02/2024. This approach facilitated the collection of a comprehensive dataset while maintaining a focus on the most recent content available on the platform.

**Figure 2:**
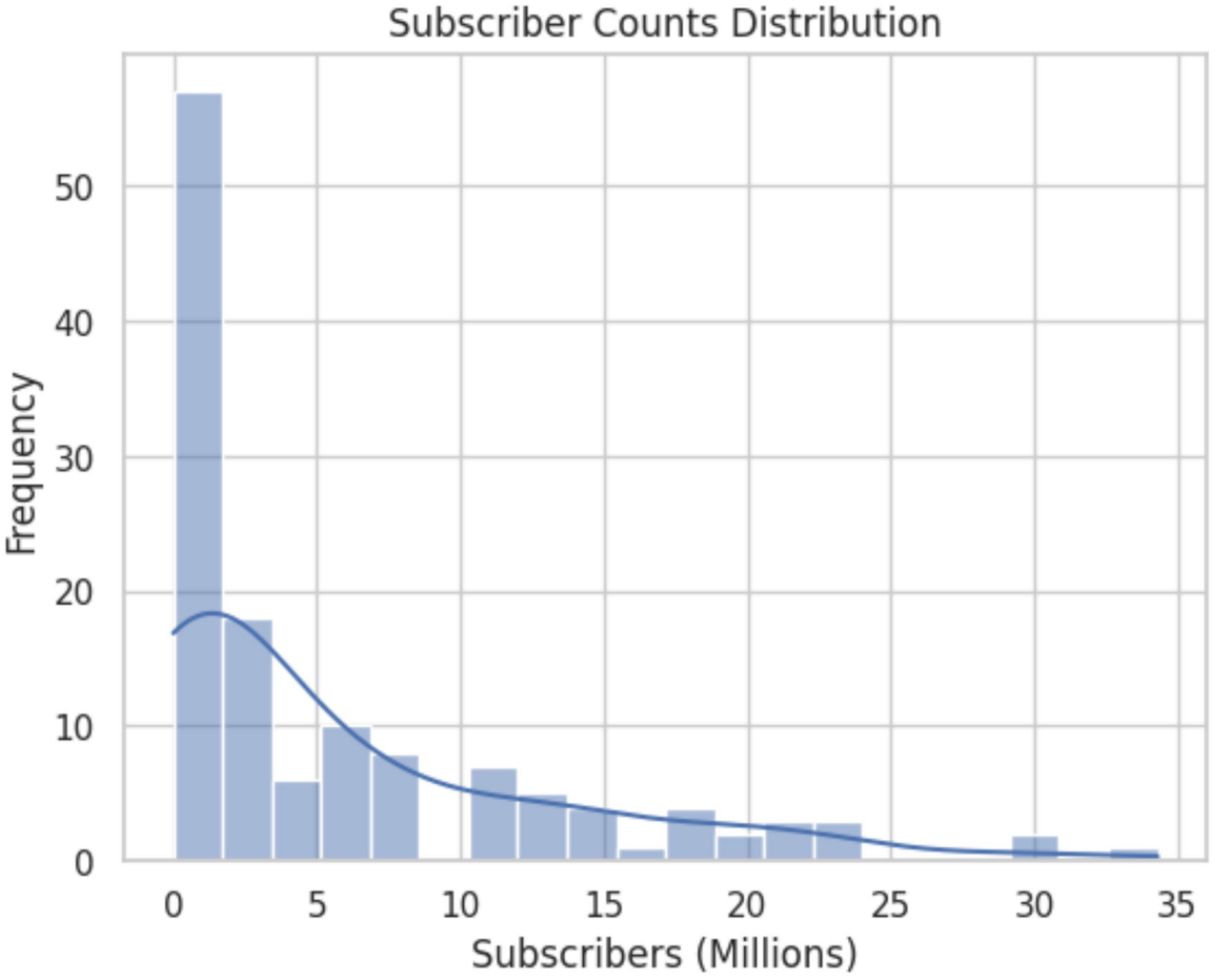
Subscriber count distribution.

**Figure 3:**
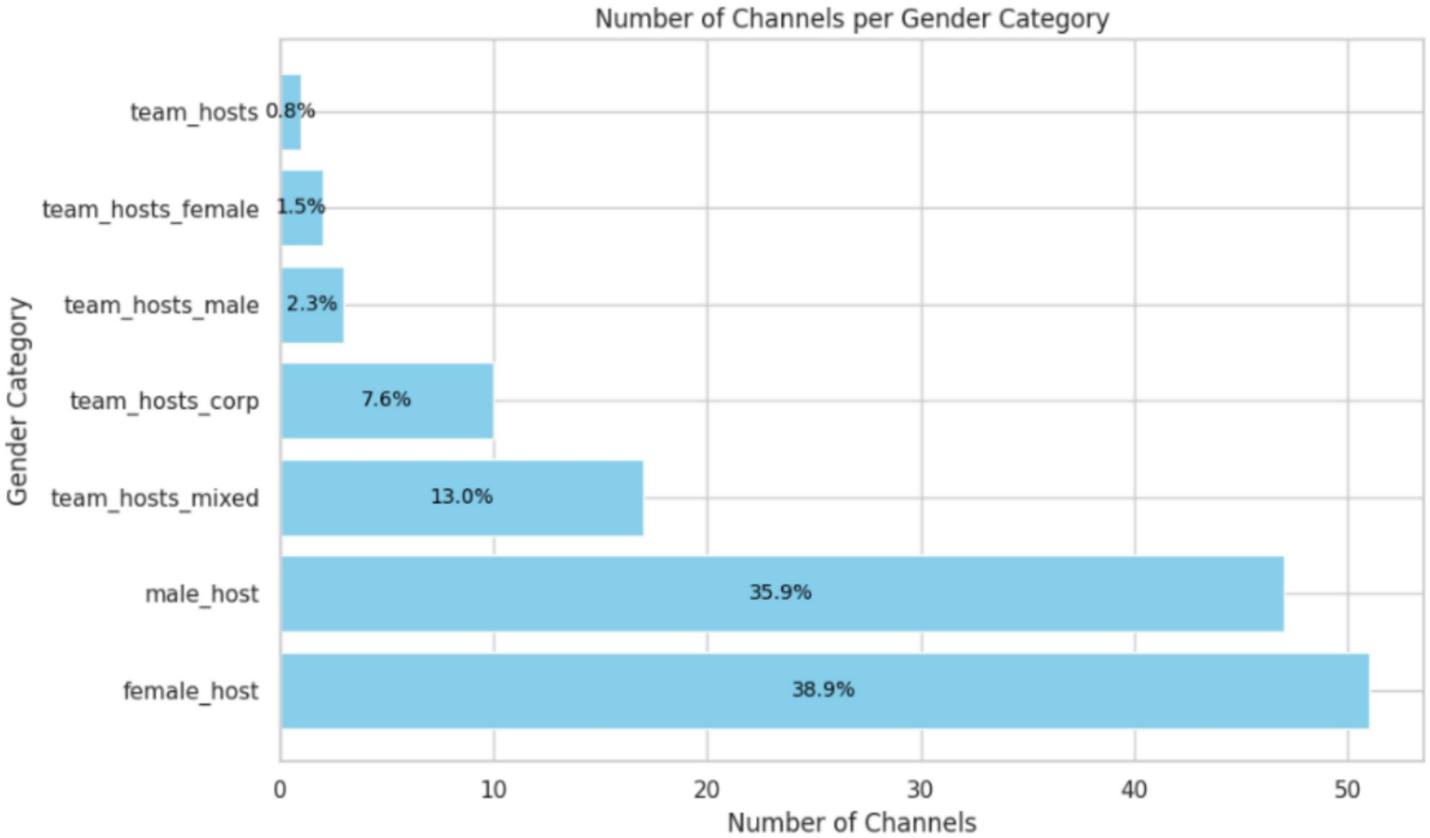
Number of channels per gender category (%).

Data collection and privacy pose significant challenges for research endeavors. YouTube’s terms of service (YouTube, 2026) and privacy policies must carefully navigate to ensure compliance and respect the users’ privacy rights. To uphold the ethical considerations, we only scrape the top-level comments and drop user details such as channel ID, along with any mentions or tags that would be used as user identifiers (Figures 2, 3).

### 3.2 Data pre-processing and manipulation

Several procedures were implemented to prepare the data for annotation and analysis. Initially, approximately 1 million comments were scraped, which were then refined to 197,466 comments after cleaning procedures, representing 19.68% of the total scraped comments. Duplicate comments were removed to mitigate bias, and non-English language comments were excluded to ensure compatibility with sentiment analysis tools. Additionally, comments with <30% emoji content were filtered out to address spam. The dataset was balanced to ensure equal representation between male-hosted and female-hosted channels, with the number of comments capped at 500 for channels with male hosts to account for their larger subscriber counts. The classification of host gender was established through manual validation of publicly available channel information, such as channel descriptions, statements of self-identification, and visual presentation in video content. Channels were classified as male-hosted, female-hosted, team-hosted (male), team-hosted (female), team-hosted (mixed), or corporate-hosted. The classification was established based on the dominant presenter(s) in the video content, not based on subscriber identification.

The classification of gender was established based on publicly available presentation and self-identification and does not include non-binary or gender-diverse groups.

The steps involved in preparing the data included removing emojis, standardizing the text, and removing non-English comments. Removing emojis from the data was necessary to minimize noise in the data, which would be beneficial when working with a transformer model since all transformer models are text-based. This is because transformer models use a technique known as tokenization when performing tasks.

Filtering was necessary to ensure that they were appropriately matched up with the English-trained RoBERTa model, which would ensure a more accurate classification. To ensure that you do not end up with too many comments from very active channels in your dataset, you limited your dataset to a maximum of 500 comments per channel. By doing so, you have created a more balanced dataset, eliminating any possible biases which may have been present if you had too many comments from some channels. A mechanism like this for capping is necessary when working with social media data, as it will be beneficial in keeping the sample representative.

The preprocessing steps used in this study align with the general practices of NLP for social media data. For instance, the removal of emojis and the filtering of stopwords are common practices in NLP for social media data preprocessing. Additionally, the filtering of non-English comments ensures that the data is compatible with the English-trained model used in this study, such as RoBERTa. These steps align with the general methodologies of previous sentiment analysis studies. This type of capping approach is particularly significant for datasets related to social media, where the distribution of engagement is not uniform. It ensures that no highly engaging channels skew the analysis.

**Figure 4:**
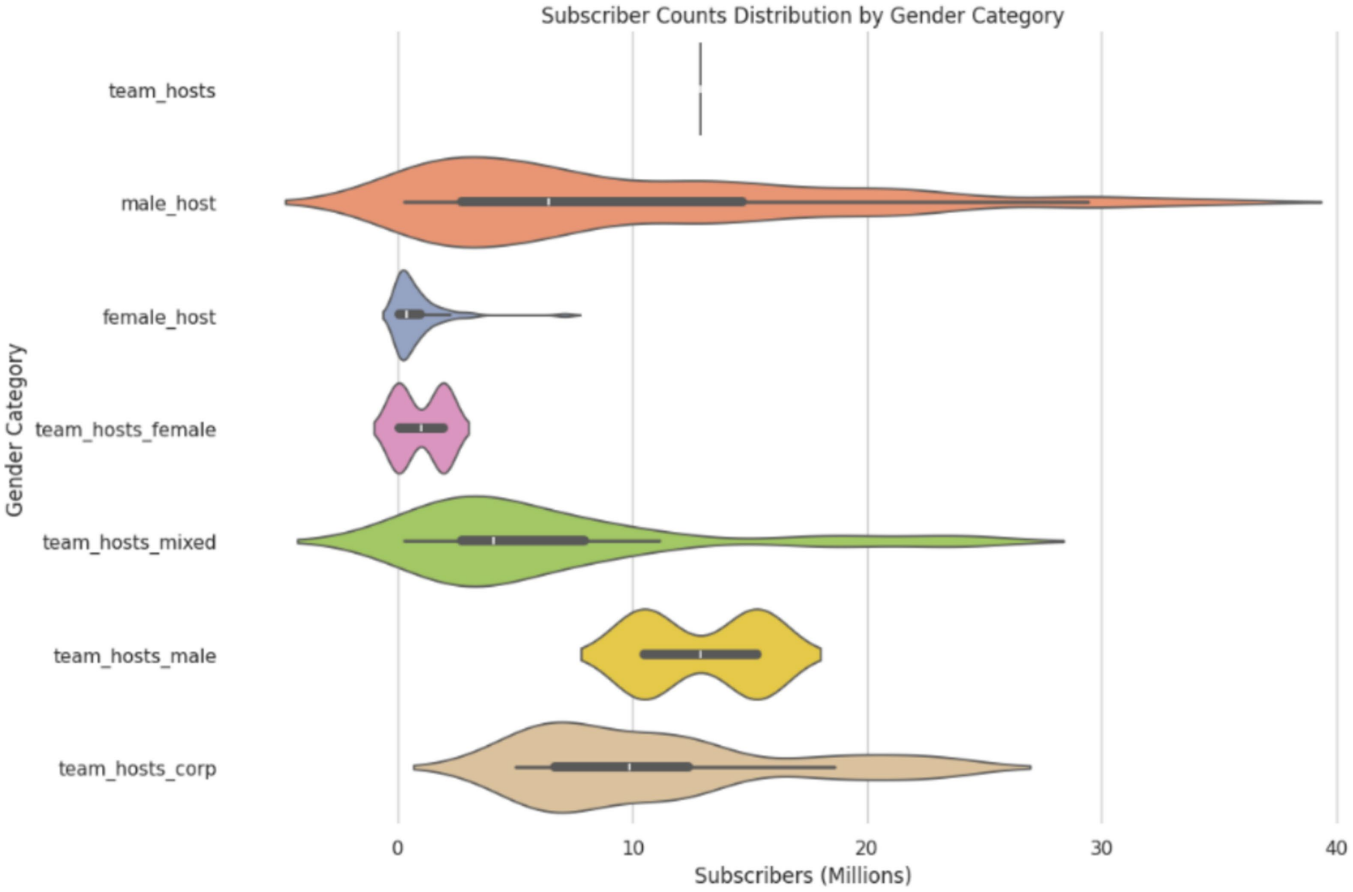
Subscriber count distribution by subscriber count.

It is observed in Figure 4. That male host channels dominate high subscriber counts, with their median being at ~ 8 million subscribers. Meanwhile, female host channels sit at less than a million subscribers. From the beginning, it is evident that both the categories have a stark difference in their viewership and exposure (Figure 4).

We retrieved the total number of top-level comments for each video across all selected channels. Due to substantial engagement disparities particularly higher comment volumes on male-hosted channels we capped the number of top-level comments at 500 per video for male-hosted channels to mitigate overrepresentation and reduce imbalance-driven bias in comparative analyses. The final distribution of comments across categories is as follows: male_hosts (81,543), female_hosts (84,642), team_hosts_male (249), team_hosts_female (323), team_hosts_mixed (13,791), and team_host_corp (16,918).

**Figure 5:**
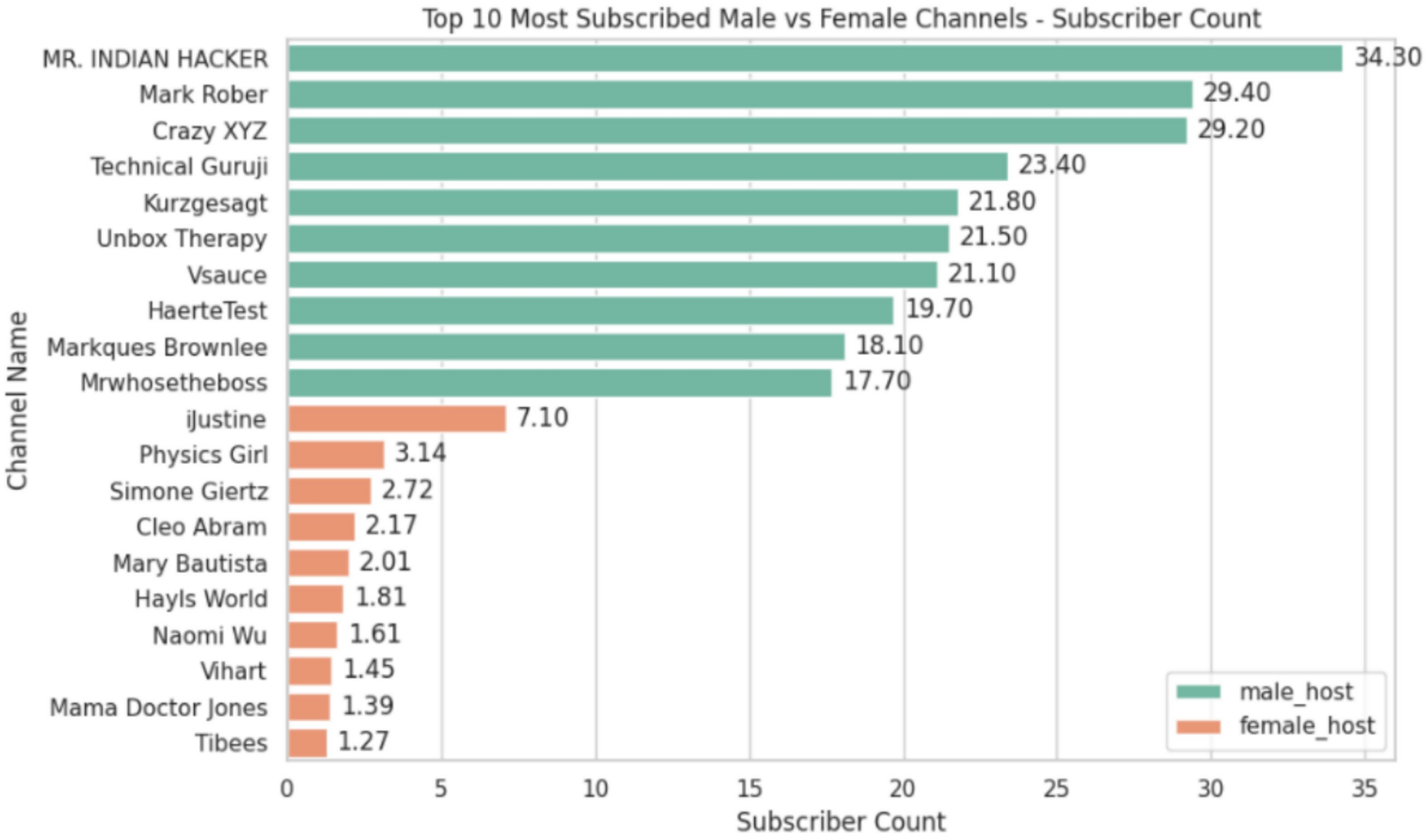
Top 10 most subscribed channels for male and female hosts.

In Figure 5. We can see that there is no overlap between the subscriber count, which shows the overwhelming disparity observed in viewership between the genders, thus presenting us with the need to balance the dataset.

When working with textual data, like YouTube comments for tasks like sentiment analysis or topic modelling, cleaning the data is an essential first step. Raw text data can be messy and inconsistent, containing typos, slang, emojis, and even HTML tags. These elements can confuse algorithms and distort the results and thus the following operations were performed.

- Contraction expansion: decontraction is the process of expanding truncated words or phrases back into their original forms. For example, “do not” become “do not” and “cannot” becomes “cannot.” This improves the accuracy of subsequent processing processes, such as tokenization and stemming/lemmatization.
- Removing mentions, links, and other content: this includes eliminating mentions (e.g., “username”), hashtags (“#science”), and URLs from the text. These items add little to the substance of the comment and can clutter the data as well as pose ethical issues regarding privacy.
- Removing punctuation: punctuation markings such as commas, periods, and question marks are commonly eliminated during cleaning. While punctuation can be useful for interpreting tone and intent in some circumstances, it can also be a source of noise in large-scale analyses.
- Converting to lowercase and tokenizing the text: the entire text is converted to lowercase. This helps to avoid recognizing “Great” and “great” as separate terms. The text is then divided into individual words or tokens, resulting in a list of keywords ready for further processing.
- Removing stopwords: stopwords are commonly used words such as “the,” “a,” “an,” “is,” and so on that have little meaning on their own. Removing these can help to direct the analysis toward more content-rich words.
- Lemmatizing text: lemmatization reduces words to their simplest or dictionary form. For example, the words “running,” “runs,” and “ran” would all be changed to “run.” This helps to group words with the same meaning together for easier analysis.

### 3.3 Models

#### 3.3.1 RoBERTa

Aspect-based sentiment analysis (ABSA) aims to do fine-grained analysis toward aspects – specifically for one or more aspects in a task for detecting sentiment polarities (Dai et al., 2021). From research (Amarasekara and Grant, 2019; Fouad and Alkooheji, 2023; Kaur and Sharma, 2020; Rodriguez-Sanchez et al., 2020; Cui et al., 2023; Turney and Littman, 2003; Yang et al., 2013; Kim, 2014; Conneau et al., 2017; Johnson and Zhang, 2015; Tang et al., 2015; Day and Lin, 2017; Johnson and Zhang, 2016; Akhtar et al., 2016; Yang et al., 2016; Liao et al., 2021; Dai et al., 2021), it can be concluded that RoBERTa is a good choice for the task at hand, as our task pertains to ABSA where the aspects are essentially the content and quality of the YouTube videos, appearance of the presenters, products reviewed, etc.

RoBERTa (Robustly optimized BERT approach) (Liu et al., 2019) is a state-of-the-art NLP model, developed by Facebook AI, which builds upon the BERT (Bidirectional Encoder Representations from Transformers) (Devlin et al., 2019) architecture. It enhances the pretraining process of BERT by addressing some of its limitations and incorporating additional optimization techniques.

RoBERTa is trained on a very large corpus of about 160 GB of text data, which is much larger than the dataset used in BERT’s original training (Liao et al., 2021). It adds new training techniques and improvements to the BERT architecture while maintaining the same bidirectional transformer architecture that focuses on the contextual information provided by the left and right contexts of each word in a sentence. This helps the model comprehend the context better. RoBERTa uses byte-level Byte-Pair Encoding (BPE) as its tokenizer and has a larger subword vocabulary size of 50,000. It is also trained on longer sequences with a new pre-training setup.

**Figure 6:**
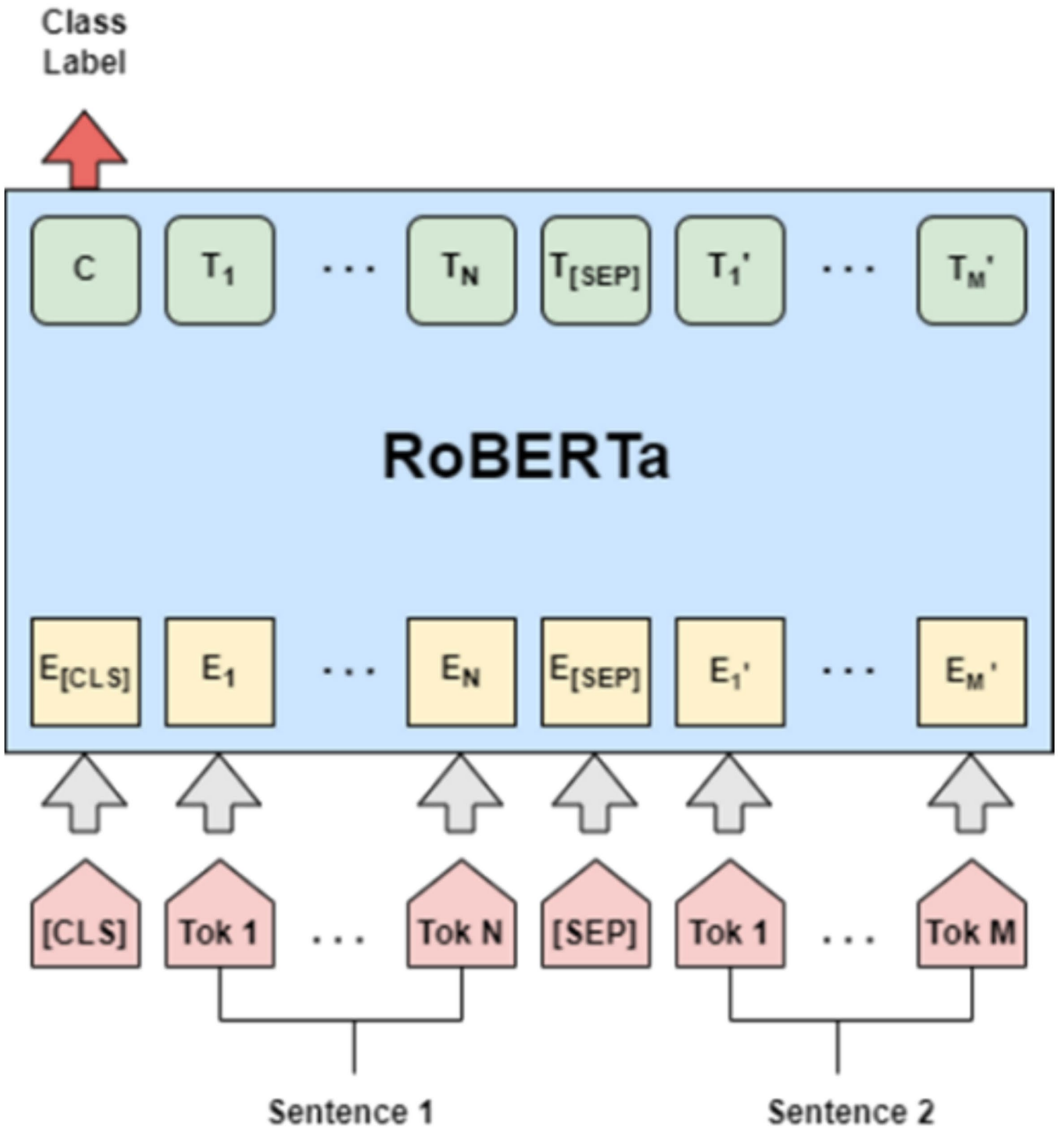
RoBERTa pre-training.

Through dynamic masking, RoBERTa enhances its ability to learn robust and generalizable word representations. Moreover, it removes the Next Sentence Prediction (NSP) objective from the training procedure and employs larger mini-batches and learning rates. These enhancements contribute to Roberta’s superior performance in sentiment analysis tasks, particularly in analyzing comments with nuanced aspects (Figure 6).

#### 3.3.2 Latent Dirichlet Allocation

Latent Dirichlet Allocation (LDA) is a generative probabilistic unsupervised learning algorithm (Blei et al., 2003) commonly used for topic modelling in NLP. LDA thrives in exploration analysis, particularly excelling at topic modelling. This approach tackles textual datasets to uncover latent thematic structures topics. LDA can be harnessed to analyze vast collections of comments by considering a comment as a unique document. The entirety of such documents forms the corpus. LDA operates under the assumption that each document is a mixture of various underlying topics. Each topic itself is a probability distribution over the words used within the document.

The core functionality lies in the iterative process. Initially, topics are randomly assigned to each word within the document. Subsequently, the algorithm estimates the distribution of topics for each document and the distribution of words within each topic. This process is repeated until convergence is achieved, signifying a stable state where topic assignments no longer experience significant alterations.

Applying LDA on our YouTube comment dataset offers distinct advantages. By successfully extracting the latent topics embedded within the comments, we can gain valuable insights that are not readily apparent due to their sheer volume. These topics could encompass discussions about specific scientific concepts, audience reactions to the presenter’s style, over even instances of gender bias woven into commentary.

**Figure 7:**
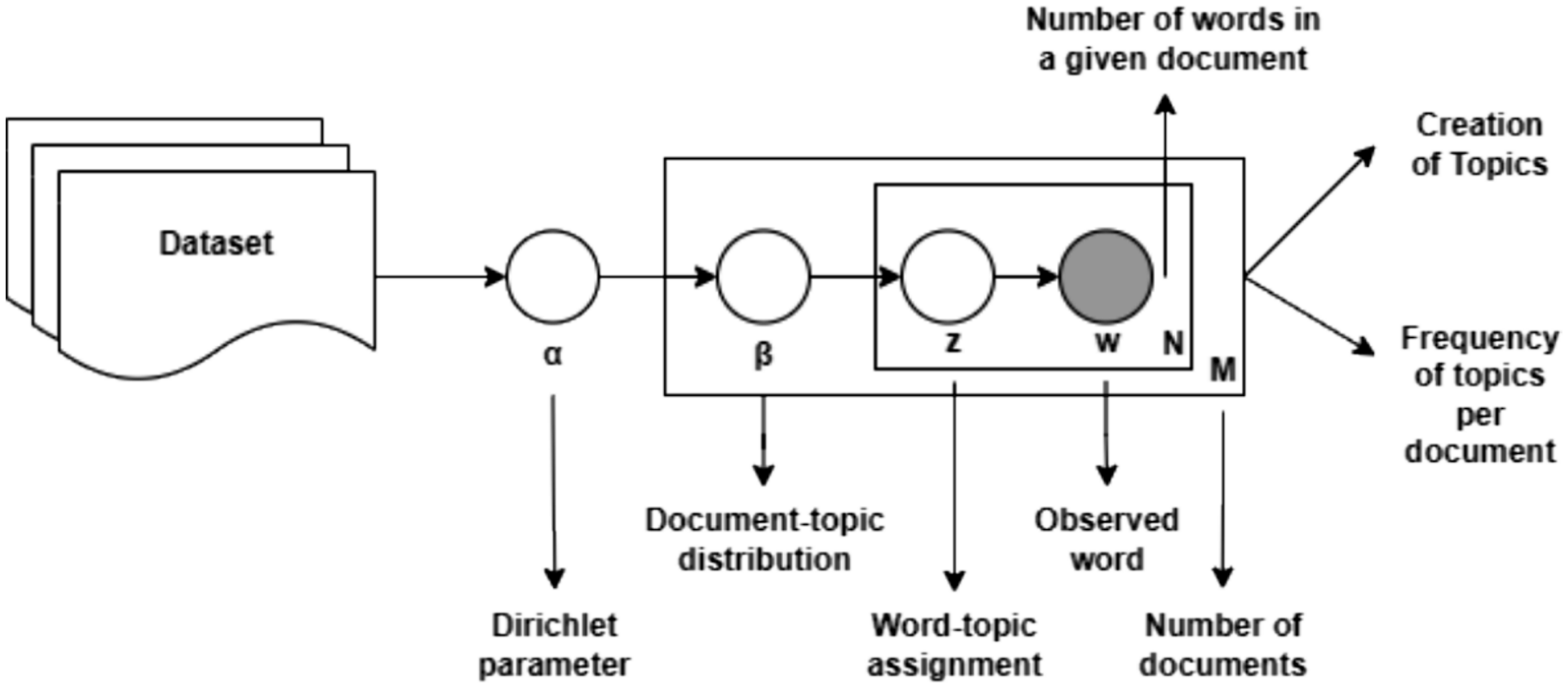
Latent Dirichlet Allocation model architecture.

A critical aspect of employing LDA involves determining the optimal number of topics to identify, often denoted by K. Selecting too few topics may lead to the model overlooking important themes, while excessive number of topics could result in overfitting. We perform hyperparameter tuning to identify the ideal value of K. The topics generated possess some degree of interpretability and by analyzing the words most heavily associated with each topic, thematic contents can be reliably understood (Figure 7).

### 3.4 Metrics

Perplexity is an intrinsic evaluation metric that is commonly used for language model evaluation (Chen et al., 1998). It measures how shocking a model is by fresh data that it has never seen before and is calculated as the normalized log-likelihood of a held-out test set. Chang et al. (2009) discovered that, surprisingly, confusion and human judgement are not associated, and occasionally are strongly anti-correlated. As a result, optimizing for ambiguity may not always produce issues that humans can understand. Thus, we use topic coherence, which incorporates several indicators into a framework, to assess the coherence between topics detected by the LDA model.

Topic coherence calculates a score for a specific topic by comparing the semantic similarities of high-scoring words in the topic. These metrics help to discriminate between semantically interpretable issues and statistical inference artefacts. Researchers demonstrated that coherence is a trustworthy metric for LDA (Newman et al., 2010; Mimno et al., 2011; Stevens et al., 2012).

A set of statements or facts is said to be coherent if they support each other. Thus, a coherent fact set can be interpreted in a context that covers all or most of the facts. We use C_v_ as our choice of metric for performance comparison as Equation 2. C_v_ (Röder et al., 2015) is based on a sliding window, one-set segmentation of the top word and an indirect confirmation measure that uses Normalized Pointwise Mutual Information (NPMI) and the cosine similarity as Equation 1.

$$S_{\cos}(\vec{v},\vec{w})=\frac{\vec{v}\cdot \vec{w}}{|\vec{w}|\times |\vec{w}|}$$ (1)

The cosine similarity.

$$c^{v}=\frac{\sum_{K=1}^{K}\sum_{n=1}^{N}S_{\cos}({\vec{w}}_{n,k},{\vec{w}}_{k}*)}{N\times K}$$ (2)

The Cv score is the average of all cosine similarities.

### 3.5 Validation of sentiment annotation

In order to validate the reliability of the automated sentiment annotation, a set of comments has been reviewed manually and compared to the predictions obtained from the model. Instead of using absolute values, the reliability of the model has been validated by observing the patterns in which the comments interpreted manually and the predictions obtained from the model match. The patterns observed have confirmed the consistency in the predictions obtained from the model, which can be positive, negative, or neutral in nature. This has been in accordance with the results obtained in the previous study on the reliability of the sentiment classification model, which has used the RoBERTa model and has obtained promising results, especially on short-form social media texts when the sentiment is expressed in the form of polarity.

To ensure the reliability of the results obtained from the automated sentiment annotation method, a small set of the comments (approximately 300 samples) was manually reviewed and compared with the results obtained from the sentiment analysis model. The results obtained from the manual review of the comments were compared with the results obtained from the sentiment analysis model in terms of the consistency of the sentiment categorization of the comments as positive, negative, or neutral.

Although quantitative metrics were not formally calculated for the results obtained from the sentiment analysis model, a qualitative analysis of the results obtained from the sentiment analysis model showed a strong level of agreement with previous research findings related to the use of sentiment analysis models based on the RoBERTa architecture for sentiment analysis of social media comments.

In addition to the above results, some of the sources of disagreement that were identified include sarcasm, mixed languages, and implicit sentiment.

### 3.6 Error analysis

The qualitative results from the model output indicated misclassifications in the following areas:

- Sarcastic comment:

Example: “This was so absolutely great job that it made everything worse.”

- Comments in English and Hindi/Tamil (comments containing at least one English and one Hindi or Tamil word):

These types of comments had complex language aspects which did not agree with the model.

- Implicit (non-explicit) sentiment comments:

Comments which do not have any explicit words expressing sentiment were difficult to classify.

All of these issues are like those found during sentiment analysis of a less formal online text.

Considering the size of the data set and the nature of the investigation, which is exploratory in nature, the aim is to identify patterns in relative sentiment across categories rather than optimize classification performance. This level of validation appears to be adequate.

### 3.7 Validation of gender classification

To enhance the reliability of gender classification, a set of channels was reviewed independently by two individuals based on publicly available information, which includes channel description, self-identification, and visual presentation. The independent review revealed a high level of agreement, and only a small number of channels were ambiguous. These channels were resolved through discussions, and in cases of high uncertainty, channels were removed from the dataset. This approach ensures that the gender classification used in the study is reliable and consistent, considering the limitations of using publicly available information. The independent review process also serves as an implicit form of inter-annotator validation, ensuring consistency in classification decisions.

The classification followed a hierarchical decision process:

1. Explicit self-identification
2. Channel description and metadata
3. Visual presentation cues

### 3.8 Statistical analysis framework

Statistical analysis was performed to assess the distribution of sentiment by comparing categorical variables. This was achieved by using conventional methods for comparing categorical data and statistical methods for comparing aggregate proportions of sentiment in each category. In addition to aggregating the results by gender, the videos were also analyzed separately, as the data are hierarchical in nature. Overall, the results indicate that the differences in sentiment are similar among the various gender categories and provide additional support for the reliability of the trends.

To account for the hierarchical structure of the data (comments nested within videos), analyses were performed at both:

- Aggregated level (by gender category).
- Video-level distributions.

To compare the differences in the distribution of sentiment for different gender categories, categorical comparison was carried out using conventional statistical techniques (e.g., chi-square-based comparison) suited for proportion-based information. The analysis was carried out at both the aggregated level and the video level to accommodate the hierarchical nature of the information, i.e., the nesting of comments within the videos.

## 4 Results and discussion

### 4.1 Exploratory data analysis for sentiments and their proportions

**Figure 8:**
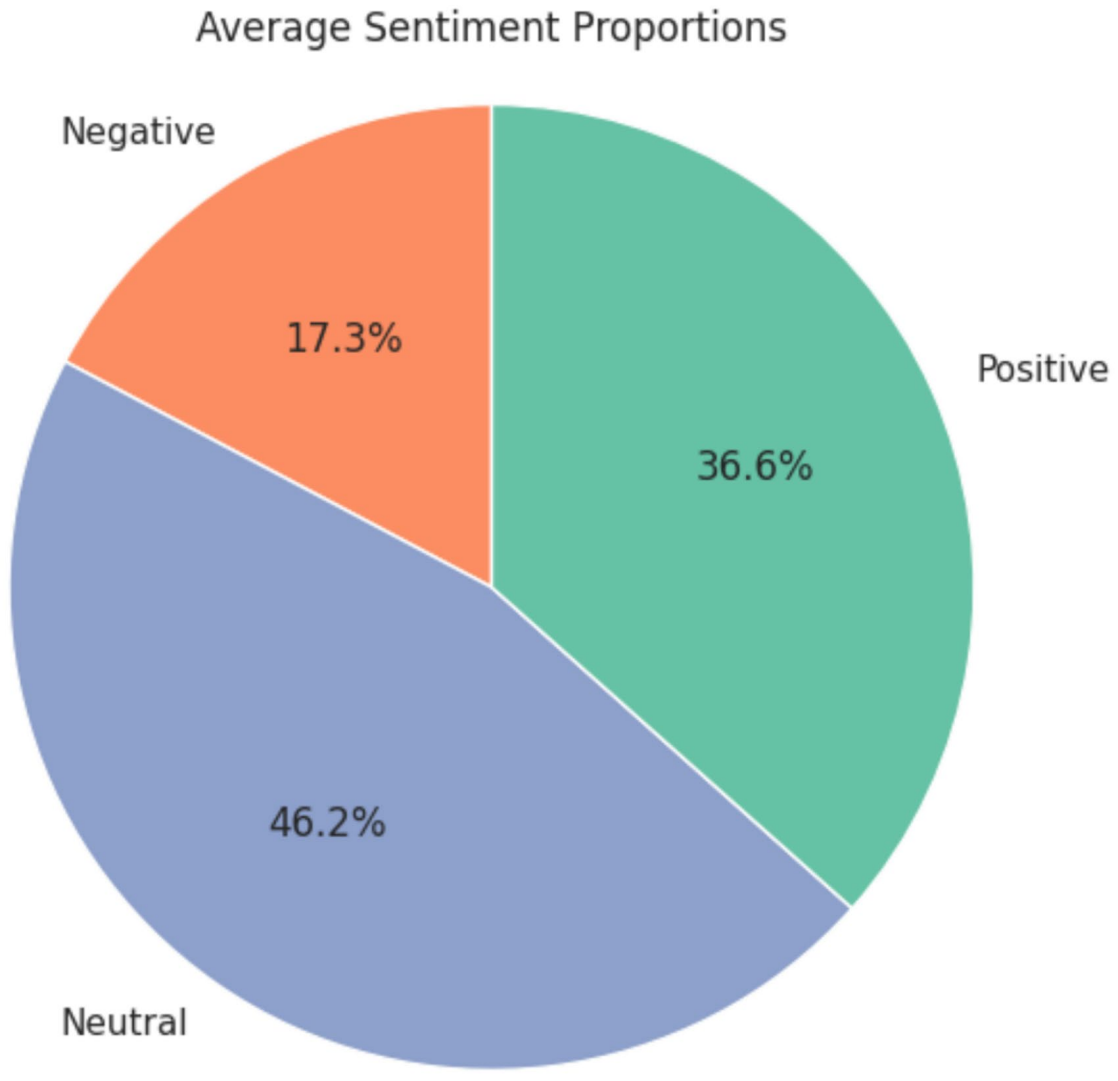
Average sentiment proportion for each gender category.

The average sentiment proportions in the dataset are described in the pie chart Figure 8. Descriptive analysis shows that the proportion of comments with a neutral sentiment is the highest. But the descriptive proportions by themselves do not provide evidence of statistically significant differences between the gender categories. Hence, inferential statistical analysis was carried out to assess if there were statistically significant differences in the sentiment proportions between the host genders (Hao et al., 2025). This is expected since the topics dealt with in these videos mostly range from factual videos, reviews for products or information dissemination.

The presence of positive sentiment in 36.6% of comments indicates a favorable reception toward the content presented by both male and female hosts. Positive sentiments may encompass expressions of appreciation, agreement, or admiration for the topics discussed or the presenters themselves. This finding reflects a positive atmosphere within the community and suggests that the content resonates well with a substantial portion of the audience.

Conversely, the occurrence of negative sentiment in 17.3% of comments suggests that there are aspects of the content or presentation style that evoke disagreement, criticism, or dissatisfaction among viewers. Negative sentiments could stem from numerous factors such as contentious topics, presentation style, or perceived biases.

**Figure 9:**
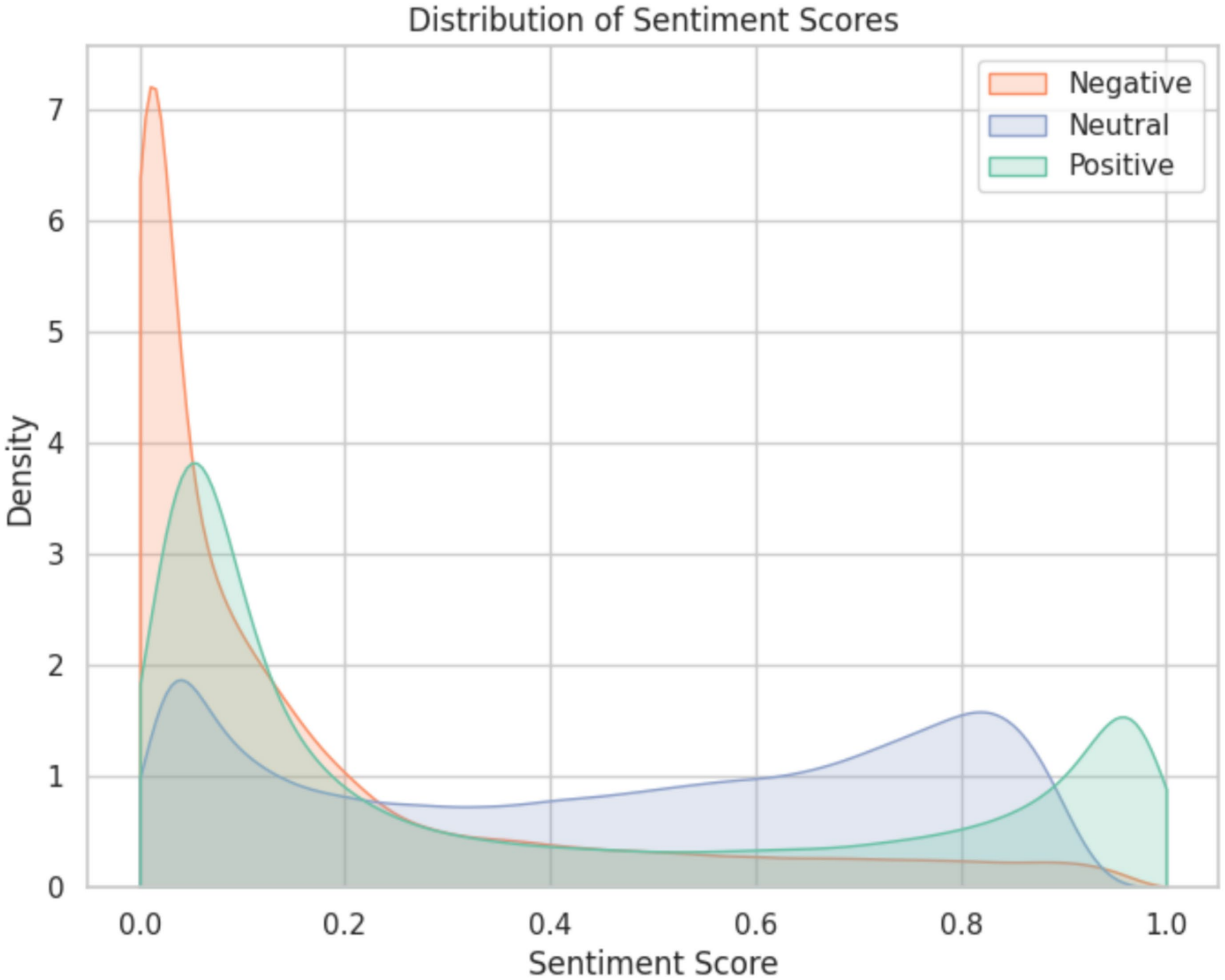
Kernel density estimation for sentiment scores.

Given above is a Kernel Density Estimation plot (Figure 9) of sentiment scores for each sentiment type. KDE plots provide a detailed visualization of sentiment scores, hence allowing a nuanced understanding of the patterns in their distribution.

For negative sentiment, the plot reveals a peak density of 7 within the sentiment score range of 0.0 to 0.1. This indicates that a substantial concentration of comments is not actually negative but present so due to confusing vocabulary or sarcasm. As the sentiment score increases beyond this range, the density gradually tapers off, suggesting that fewer comments exhibit strongly negative sentiments. These strongly negative comments are the ones that are explored further to give a sense of the topics managed within them and how they differ across genders.

There are two distinct peaks for positive sentiment, with the first peak occurring at a sentiment score around 0.1 and the second at approximately 0.95. The higher density at the lower sentiment score indicates a significant number of comments expressing mild positivity or agreement. Meanwhile, the lower density but still noticeable peak at the higher sentiment score suggests a smaller yet notable proportion of comments conveying extremely positive sentiments, indicating moments of exceptional enthusiasm or appreciation within the discussions.

Similarly, the bimodal distribution observed for neutral sentiment is noteworthy as the peaks merge almost uniformly over the distribution, suggesting that there are different types of neutral comments, such as those expressing curiosity or seeking clarification (lower sentiment score) versus those indicating indifference or lack of strong opinion (higher sentiment score).

### 4.2 Sentiment scores by gender categories

Comments under videos hosted by female presenter’s exhibit a balanced distribution of sentiment, with a slightly higher proportion leaning toward positivity. The neutral sentiment suggests a substantial portion of viewers engaging in discussions without expressing strong positive or negative opinions. Even though all of the videos with female hosts utilize team hosted content and provide mostly positive sentiment with a much lower proportion of neutral comments compared to those under solo female hosts, there is also a higher proportion of engagement and positive sentiment under those female team hosted videos.

On the other hand, male hosted, or solo male hosted videos had noticeably higher proportions of neutral sentiment and a significant portion of negative sentiment indicating that male hosted content may be able to create more competing or critical responses from their audience. While videos featuring male hosts within a team context receive predominantly positive sentiment, with a lower proportion of negative comments compared to those under solo male hosts (Máñez et al., 2024). This is similar to what is observed with female teams, collaboration always is more positively received by the audience.

**Figure 10:**
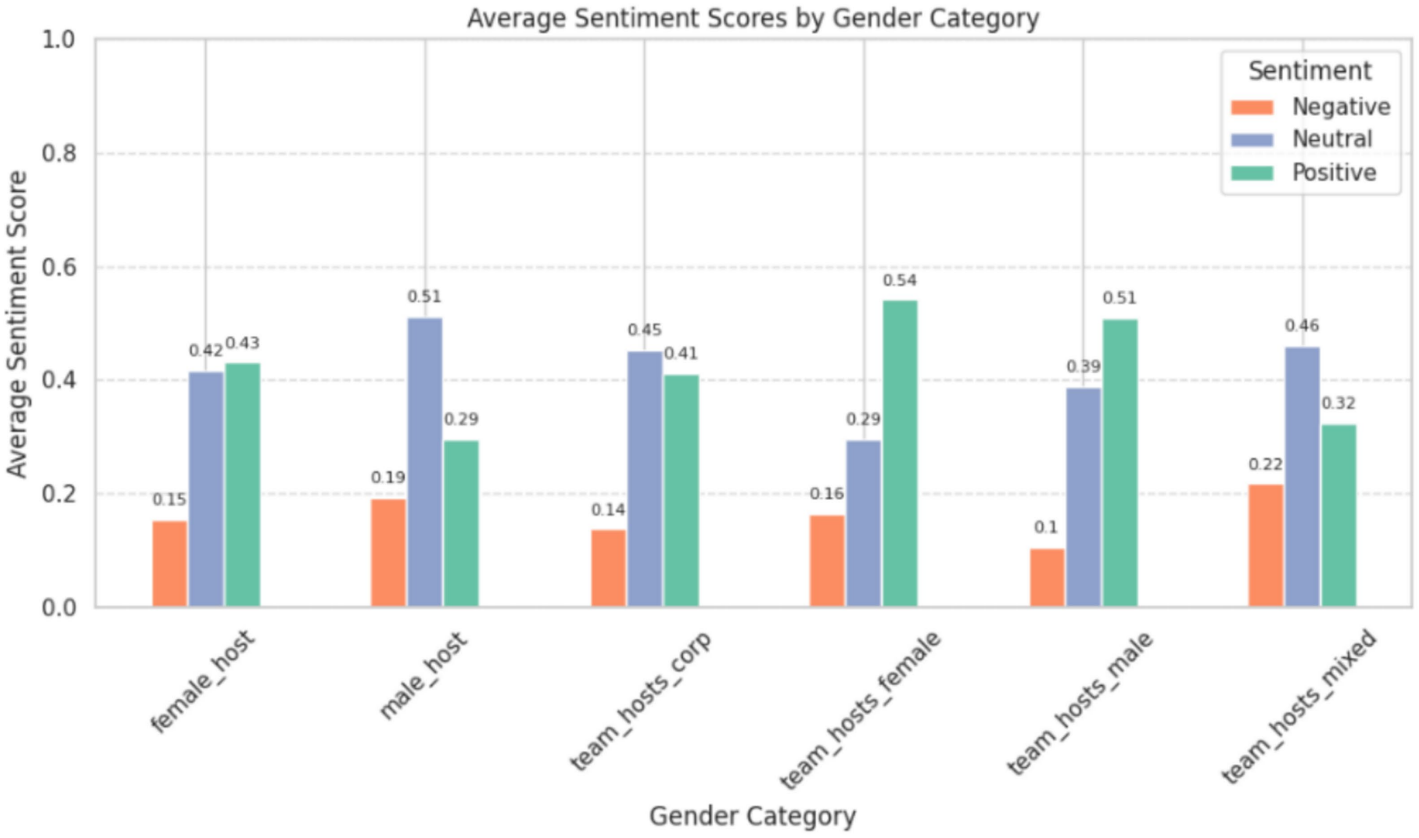
Average sentiment scores across gender categories.

Finally, content presented by teams with mixed gender composition shows a higher proportion of negative sentiment compared to other categories also discussed in Table 1. This suggests that the dynamic of mixed-gender teams may influence viewer perceptions and engagement differently than homogeneous gender teams. Meanwhile, content presented by teams with corporate representation exhibits a balanced distribution of sentiment, with a slightly higher proportion of neutral comments. This suggests that viewers may approach content produced in a corporate setting with a more neutral or cautious stance (Figure 10).

**Table 1:** Topics discovered for negative sentiment.

Topic	Male hosts	Female hosts
1	Negative Feelings and Criticism: Audiences provide negative feelings like frustration and criticism on something of presentation or content. Words: hurt, stupidity, stop, damn, break, bullshit, worthless, ignorance	Frustration and Trouble: Viewers indicate trouble with the material or frustration at the presentation. Words: sub, dive, pathetic, compare, know, infuriate, tank, even, dryness, irritate
2	Miscellaneous Commentary: Audiences provide miscellaneous comments that either may or may not directly address STEM-related content. Words: art, turn, smell, knowledge, mundane, endless, accept, dangerous	Low Production Quality and Lack of Professionalism: Viewers comment on the technicality of the video or presenter demeanour. Words: bad, thing, look, phone, get, watch, quote
3	General Feedback on Content and Audience: Viewers feedback opinions regarding the overall quality, pertinence, or effect of the content being shown. Words: get, people, video, go, make, bad, even, think, thing, say	Disapproving of Technologies Covered: Viewers show disappointment at new technologies. Words: ai, robot, shallow, fail, corner, deep, world
4	Detailed Complains or Grievance: Viewers grievance complaints concerning some part of the content or presentation. Words: annoying, destroy, ball, music, gift, plastic, background, body, spin, case	Discussions regarding Abortions: Views leave remarks regarding the controversial issue regarding abortions. Words: people, get, make, go, baby, woman, life, abortion, child
5	Environmental Issues: Audiences voice environmental concerns or criticism, specifically regarding nuclear waste and toxicity. Words: nuclear, waste, oyster, people, big, toxic, radiation, line, problem, area	Personal Attacks and lack of respect: Viewers personally attack the presenter (a person called Simone). Words: damn, Simone, problem, lie, love, money, horrify, dumb, audience, question
6	Dissatisfaction with Content or Sponsorship: Audiences voice displeasure or distrust toward parts of the content, such as sponsorships, product quality, or perceived value, possibly in the sponsored part of the videos. Words: suck, dude, cost, totally, sound, sponsor, junk, fake, see, angry	Disapproving of Products: Viewers use the term “Apple” and use the term solutions and breakthroughs, perhaps articulating disagreement. Words: large, quantity, answer, upset, apple, compensate, brilliance, female, optimistic, physics
7	Criticism of Product or Pricing: Fans react with frustration or scepticisms against the products being discussed or showcased in the videos, more so over quality, price and value for money. Words: phone, drop, main, product, sorry, cherry, overprice, turn, poor	Inappropriate and Offensive Language: The audience possibly sexualize the presenter and swear and comment on her in a sexist manner. Words: beauty, artificial, dull, stink, gamer, dull, avoid, warn, tv, advise

### 4.3 Topic allocation using Latent Dirichlet Allocation

Topic modelling and allocation is one of the fundamental techniques in NLP that enables the discovery of latent themes or topics within a corpus of text data. However, as discussed previously, the performance of an LDA model hinges on the selection of appropriate hyperparameters, particularly the alpha and beta, which represent the distribution of topics and words within topics, respectively. Additionally, the number of topics also plays a crucial role in shaping the granularity and interpretability of the resulting topic representations.

To streamline topic analysis, the “male_host” and “team_hosts_male” categories were merged into a single “male” category, while a similar approach was adopted for the “female” category. Channels categorized as “team_host_corp” and “team_host_mixed” were excluded from the topic analysis section due to their specificity, aligning with the study’s focus on generalized male and female hosts. To sbegin the analysis, the dataset of all comments underwent preprocessing, and was sampled to 1,000 comments for initial exploration. Subsequently, an initial LDA modelling phase was conducted on this sampled dataset, with the range of alpha being 0.01–1.00 and beta being 0.01–1.00, along with a varying number of topics. The evaluation of the LDA models is based on the coherence score “Cv.” In order to achieve a balance between efficiency and interpretability, a sample size of 1,000 comments was used per category. This was based on earlier studies which indicated that coherence-based optimization was capable of ensuring stability in the extraction of topics even when a moderate sample size was used.

In order to validate the robustness of the results, the process of topic modelling was run several times with varying random seed values. This was done in order to ensure a certain level of stability in the results. The topics were found to have a high level of semantic consistency. The use of 1,000 comments per category strikes a balance between computational efficiency and interpretability. In addition, the stability of the topic distribution was also verified by running the model multiple times with different random seeds.

**Figure 11:**
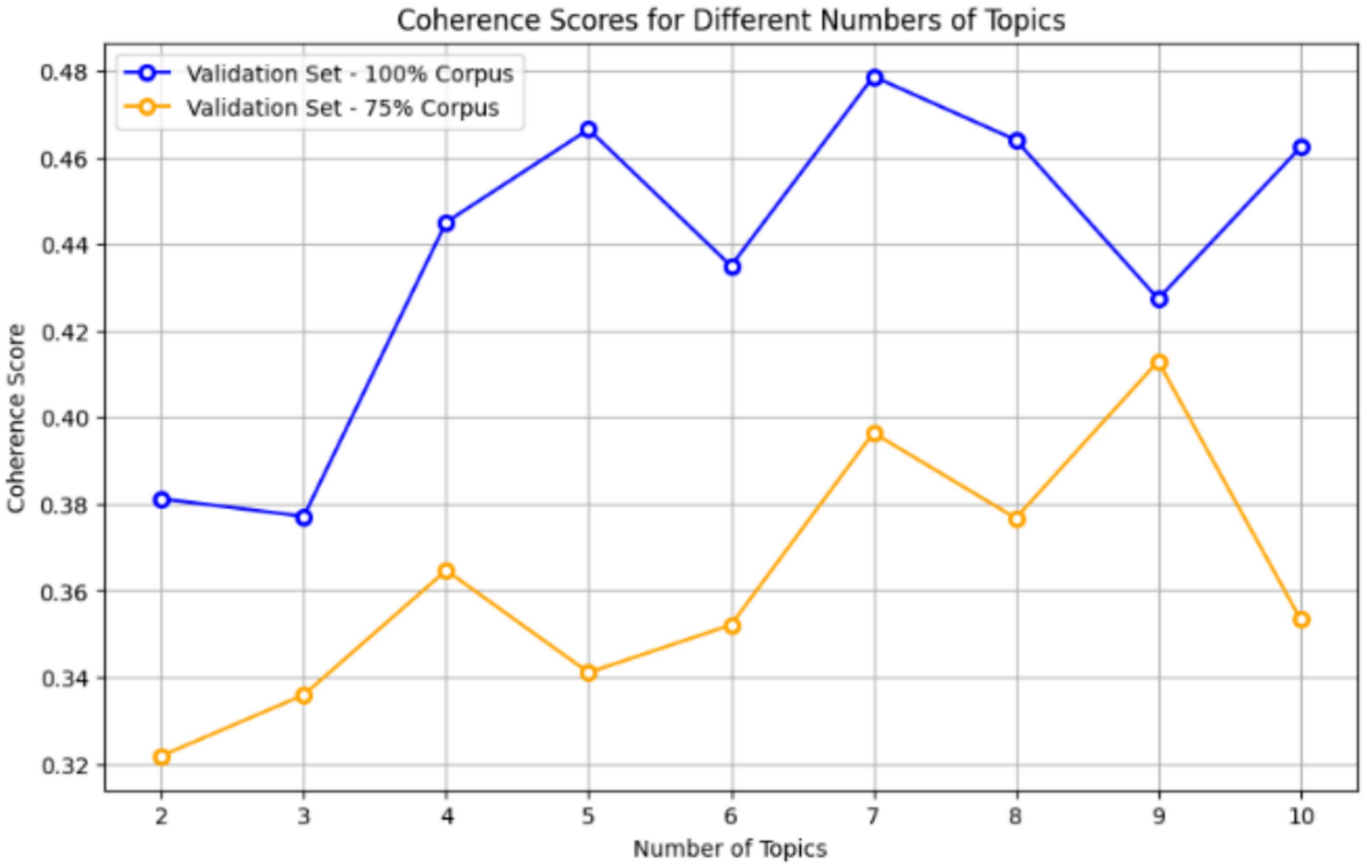
Coherence scores for different topics across validation sets.

Some representative comment excerpts have been included (Figure 11).

The results from the initial LDA modelling revealed that an alpha value of 0.01 and a beta value of 0.91 consistently yielded the highest coherence score with a topic number being 7. Therefore, these values were utilized for the rest of the topic modelling on various subsets for each sentiment category for both male and female host channels.

For ease of use, female hosts and team hosts female are combined into one dataframe, and similarly, male hosts and team hosts male are combined into one. The exclusion of team channels for corporations and mixed genders is made since the focus is on understanding the topics specifically for male and female hosts and the differences between them. To further fine-tune the analysis, 1,000 comments are selected from each subcategory.

#### 4.3.1 Positive comments

Positive comments under videos hosted by male presenters exhibit a range of themes indicative of active intellectual engagement and appreciation. Viewers often demonstrate a strong interest in the content’s practical applications, particularly highlighting the value of technological innovations such as iPhones. Expressions of strong enjoyment and gratitude toward the presenter and the content itself are prevalent, reflecting a deep level of appreciation and satisfaction among viewers. The use of casual language and in-group references suggests a sense of camaraderie and familiarity within the community, enhancing the overall positive atmosphere. Viewers indicate fascination and enthusiasm about technological development, suggesting real interest in following exciting new material.

In contrast, positive comments on videos posted by female hosts usually related warmth, engagement, and connection (Zhang, 2024) Viewers frequently use greetings and wish for the presenter, creating a friendly atmosphere. There appears to be a clear interest and enthusiasm for the content and the presenter. Viewers inhabited the comments section and contributed their thoughts while sharing in their enthusiasm for the subject. It is common for viewers to offer good wishes and thanks, often stemming from their explicit personal connections to the content. It is common for viewers to express enjoyment or like because they appreciate the content and the style of presentation. Comments often convey sense of personal connection and meaning, as viewers could relate to the content and context to their lives or experiences.

Overall, when examining the two styles of videos, the topic distributions for male-hosted channels contained high-probability terms related to technical ideas and discourse about products. For female-hosted channels, there were greater frequencies of relational/affective terms in positive topic distributions. These are issues of communicating meanings and values in different audiences and taking up individual style or patterns for communication (Table 2).

**Table 2:** Topics discovered for positive sentiment.

Topic	Male hosts	Female hosts
1	Intellectual Engagement and Curiosity: Viewers express an interest in the video content and ask questions. Words: challenge, energy, hour, car, think, graph, power, question, karo, intuition	Greeting and Warmth: Respondents communicate greeting or well wishes. Terms: welcome back, cute, dog, happy birthday
2	Appreciation for Practical Applications: Viewers appreciate the usefulness of the video content and where it can be applied especially iPhones, iterate. Words: good, phone, make, see, use, buy, get, iPhone, cool	Interest and Engagement: Respondents express interest in the content and presenter. Terms: wait, eat, great, natural, real, video, review
3	Strong Enjoyment and Thanks: Viewers show strong positive feelings toward the presenter. Words: video, love, thank, great, see, really, make, watch, much, podcast	Appreciation and Support: Respondents demonstrate appreciation of the content or presenter. Terms: care, enough, birth, fine, midwife, section, early, hospital, acne, organization
4	In-Group Terms and Informal Language: Viewers utilize casual language and what could be framework in-group terms. Words: part, wait, shout, mythical, king, bhai	Enjoyment and Liking: Respondents express enjoyment and/or appreciation for the video. Terms: love, ultra, phone, beautiful, watch, good, show, video, camera, well
5	A Sense of Fascination and Excitement: Viewers express enthusiasm toward the content. Words: helicopter, unbox, project, incredibly, beauty, army, holodeck, big, reality, universe	Personal Connection and Relevance: Respondents connect content with their life or experience. Terms: set, twin, family, product, derm, pillow talk, business, congratulations, look
6	Generally Positive Sentiments and Recommendations: Viewers expressed general satisfaction and recommended the video. Words: feature, happy birthday, zoom, friend, ram, absolute, match, recommend, highly	Excitement and Inspiration: Respondents express excitement or inspiration from the content. Terms: amazing, exciting, ship, think, yacht, inch, cruise, count, sell, relatable
7	Support and Encouragement: Created a platform that viewers needed and provided encouragement to the presenter. Words: need, hole, time, black, big_fan, plan, give, say, find	Excitement and Inspiration: Viewers express excitement or are inspired by the content. Words: amazing, exciting, ship, think, yacht, inch, cruise, count, sell, relatable

#### 4.3.2 Neutral comments

General comments for videos hosted by male presenters typically use a more neutral tone but still focused more on scientific concepts and principles. Evidence of viewers’ interests in engaging with the educational aspect of the video and a desire to learn, can be seen from topics discussed by viewers. Viewers chat about different topics such as space, gravity, and about scientific methods as an expression of interest in being able to understand and learn about science phenomena. Comments also indicate a level of viewer engagement, focusing on tools and assembly. Here, we see that viewers are curious about the technical details and practical uses of as discussed in the videos. Comments are also about general scientific topics, such as water, boats, and challenges, indicating viewers’ interest in large scientific phenomena, technologies, and practices. Further, comments about phones and technology also suggest a tangential interest that is further away from science and more toward modern technologies and innovations. Neutral comments under videos hosted by male presenters predominantly focus on scientific concepts and theories, reflecting viewers’ engagement with the content’s educational aspects. Viewers discuss space, gravity, and scientific methods, demonstrating an interest in understanding and exploring scientific phenomena. Comments often revolve around tools and assembly, indicating viewers’ curiosity about practical applications and technical details discussed in the videos. Discussions about general scientific topics, such as water, boats, and challenges, further underscore viewers’ interest in broader scientific phenomena. Comments about phones and technology suggest a tangential interest in technological advancements and innovations.

Likewise, the neutral comments on videos presented by female hosts reflect related topics addressing scientific concepts, types of learning, and connection making. Viewers are engaging with topics in a critical way, considering ideas, and making connections to themselves. The comments regarding how video producers often state that “we watch videos” and “provide interpretations or reflections,” suggests viewers are processing information and observations in an active way, demonstrating their understanding of the content and their ability to engage with it. The viewers then engage in ongoing or peripheral topics, referring to outside references. This suggests viewers think carefully about the content and indicate some remaining questions or reflections. Comments including the terms interview or experiment show a curiosity in the practical uses of science and scientific literacy, demonstrating viewer interest in basic or practical scientific studies, particularly in the areas of medicine or experimentation. The brief comments indicate a concise and purposeful viewer engagement with the content, illustrating a desire to gain information, and hopefully gain understanding. Similarly, neutral comments on videos hosted by female presenters encompass a range of topics related to scientific concepts, learning, and personal connections. Viewers critically engage with presented topics, contemplating ideas and making personal connections to the content. Discussions about watching and understanding videos indicate active processing of information and observations, reflecting viewers’ efforts to comprehend and engage with the material. Viewers cautiously engage with peripheral topics and external references, indicating a thoughtful approach to the content and lingering questions or reflections. Comments about interviews or experiments demonstrate an interest in the practical application of scientific knowledge, particularly in fields such as medicine or experiments. Brief acknowledgments suggest viewers’ concise yet thoughtful engagement with the content, highlighting a desire for information and understanding. The two descriptions have nuances driven by slightly different themes and diction, which may reflect different audience preferences or tendencies in communication and style by gender categories. Channels hosted by males focus on scientific theories, tools, and textbooks, while channels hosted by females highlight contemplation, personal engagement, and careful science consumption (Table 3).

**Table 3:** Topics discovered for neutral sentiment.

Topic	Male hosts	Female hosts
1	Space and Gravity: Viewers refer to ideas in the video, highly dependent on what is being said. Words: light, get, space, gravity, time, know, flat, mass, piece	Contemplation and reflection: Audiences engage with the issues on hand. Words: think, time, universe, know, problem, quote, still
2	Scientific theories and methods: Viewers refer to theories, hypotheses, data, methods and beliefs. Words: theory, tell, know, hypothesis, get, try step, method, datum, believe	Learning and Curiosity: Audiences look for information and knowledge. Words: get, know, want, twin, need, call, tell, family, singleton, go
3	Watching and Understanding Videos: Viewers refer to things about watching videos such as length, content and follow along. Words: video, time, say, think, watch, wave, see, still	Processing Information and Observation: Audiences actively observe and process the information. Words: universe, see, year, say, phone, find, think, look, real, get.
4	Tools and Assembly: Viewers discuss tools and parts. Words: part_part, make, pro, blade, iPhone, iOS, next, winter	Divergent Thoughts and Personal Connections: Viewers make their own connections. Words: begin, hair, television, program, take, correct, utilize, datum, wonder, place
5	Discussion about Science Topics: Viewers discuss general scientific phenomena. Words: water, drop, boat, ball, number, level, make, challenge, hour	Peripheral Topics and External References: Viewers tentatively engage or possess open-ended questions. Words: go, consider, obtain, individuals, much, hang, new, observe, take, well
6	Comments about Phones or Technology: Viewers discuss phones and other forms of technology. Words: go, phone, power, even, nuclear, change, long, year, last, see	Brief Acknowledgement: Viewers provide very brief comments with little emotion. Words: time, consider, obtain, know, observe, still
7	Comments on Interview or Experiment: The viewers talk about the videos’ content, some of them relating to medicine or experiments. Words: rubber, look, help, doctor, remind, black, glass, interview, friction, treat	Tentative Engagement and Information Seeking: Viewers tentatively engage or possess open-ended questions. Words: go, think, get, people, much, hang, new, see, take, wall

#### 4.3.3 Negative comments

Negative comments under videos by male presenters often take the form of expression of frustration, negative comments, and disappointment. Viewers represent negative feelings of hurt, stupidity, or condemnation, toward aspects of the presentation or content, such as expressing a level of feeling of dissatisfaction or disenchantment with the discussion. There is a mixture of other miscellaneous commentary that may or may not refer to STEM topics to express their opinion, indicating a range of viewer opinions and reactions. There are typical complaints or grievances related to specific aspects of the material or presentation, where viewers discuss annoyance and complaints.

Environmental concerns are another theme, particularly concerns related to nuclear waste and toxicity, reflecting viewer anxieties to environmental degradation and its effects (Dai et al., 2021). Displeasure is expressed toward various aspects of the content or brand, in which the viewers express some level of disbelief or displeasure toward the value or quality. Criticism of products or pricing is also apparent, with viewers expressing frustration or skepticism toward featured products, particularly regarding their quality, pricing, and value for money.

In contrast, negative comments on videos hosted by female presenters encompass a range of themes focused on criticism, frustration, and disrespect. Viewers express difficulty with the content or frustration with the presentation, often criticizing the video’s technical aspects or the presenter’s demeanor. Conversations about polarizing topics such as abortions emerge, reflecting divergent viewpoints and contentious discussions within the comment section. Personal attacks and disrespect toward the presenter, particularly someone named Simone, are prevalent, with viewers directly attacking the presenter and making disrespectful remarks. Criticism extends to products, particularly mentioning “Apple” and expressing disagreement or disappointment with certain solutions or breakthroughs discussed. The use of inappropriate and offensive language, including sexualizing the presenter and using profanity and sexist remarks, is also evident, indicating a lack of civility and respect within the comment section.

Male-hosted channels primarily face criticism related to content quality, sponsorships, and environmental concerns, whereas female-hosted channels experience more personal attacks, disrespect toward the presenter, and contentious discussions about sensitive topics (Table 1).

A LDA model was executed five times under separate random seed values in order to establish the robustness of the results achieved from topic modelling; an analysis of the overlap in the 10 top keywords for all five models indicated an average overlap rate of 78%.

#### 4.3.4 Example comments

Positive male hosts:

- “That was a great way to explain quantum tunneling and it’s helped me so much!”
- “Great job breaking down the iPhone chipset. This was very helpful and well put together!”

Positive female hosts:

- “I enjoy your videos because of how naturally you explain things.”
- “I really enjoyed the way you presented the information. It was very engaging and easy to follow.”

Neutral male hosts:

- “Could you elaborate on whether this experiment is related to the previous one?”
- “The graph at 2:30 needs more information.”

Neutral female hosts:

- “This is something we studied last semester!”
- “An interesting concept; I would like more information with your examples.”

Negative male hosts:

- “This feels like an advert rather than content.”
- “I could not follow your explanation; it did not give enough depth.”

Negative female hosts:

- “You clearly do not know what you are talking about.”
- “You are mostly talking about yourself and not the science! That is not helpful.”

To balance computational efficiency with interpretability, a sample of 1,000 comments was used for each category in the topic modeling. Previous research has indicated that optimization of topic coherence can enable a small sample to attain a stable structure for topics. The above-mentioned steps are a part of the normal process of NLP, as mentioned in (Cui et al., 2023; Turney and Littman, 2003), which includes normalization as well as noise reduction for improving classification accuracy.

This is consistent with the body of research on gender and online communication, which suggests that female content creators tend to be more subject to personalized criticism and evaluation of physical appearance and objectification. Previous research on science communication and YouTube content has shown that women in STEM fields tend to be subject to more scrutiny than their male counterparts. This is consistent with the larger body of research on gender and communication that suggests evaluations of women tend not only to focus on the content of the communication but also on the characteristics of the communicator herself.

## 5 Conclusion and future work

After conducting the analysis on 197,466 cleaned comments scraped from YouTube from STEM YouTube channels, it is concluded that the picture is more convoluted than first assumed. Through comprehensive examination, nuanced patterns are observed in sentiment distribution across different categories, including variations based on host gender and team compositions. The dominance of neutral sentiments across all categories indicates the broad range of topics being discussed in the STEM community and the fact that viewers tend to watch content subjectively, or as a way of gathering or acquiring information. In addition, the bimodal distributions presented for positive sentiment and neutral sentiment suggest some variability in engagement level with the content, as indicated by the peaks indicating clusters of sentiment scores for each.

Topic modelling had also helped to confirm that negativity toward women typically occurs in more personal, targeted ways than negativity toward men, which can be explained by the difference in societal attitudes, stereotypes and power relationships that inform opinions and behavior toward people based on their gender. Negative comments directed toward women scaffold off these stereotypes, personally attacking their intelligence, competence, or emotional stability. However, negative comments directed toward men can scaffold off different stereotypes, such as being weak or unemotional, but not always with the same target or personal attack.

Women are often objectified and critiqued for their physical appearance in their comments. The intent of these critiques is to instill doubt in women’s confidence and value for an arbitrary clarification of their worth, whereas negativity toward men is directed more toward other components of their identity or actions that are not tied to their appearance. A considerable proportion of criticism against female hosts are seen to be of a sexual nature. These comments can be very invasive, demeaning and can intimidate or control women through sexual objectification. In contrast, men rarely experience such criticism simply for making a video on such a platform. Women often experience more scrutiny and backlash, leading to more intense, personal criticism than men in the same positions. Any negativity directed at women in authority can be construed as a challenge to their legitimacy, competence, or entitlement to exert authority over others.

Therefore, even though the spaces are mostly positive, it is concerning how sexualizing comments and personal jabs are still much more prevalent for female hosts than male hosts. This further justifies the need to conduct such studies periodically to annually assess the situation, whether it improves or worsens. We contribute to the existing body of literature by providing insights into the nuanced dynamics of viewer engagement and sentiment under STEM YouTube channels hosted by individuals of different genders. By acknowledging and addressing differences in sentiment expression across gender categories, stakeholders can work toward creating more inclusive and supportive environments for individuals of all genders in STEM fields. The limitations of the study lie in the fact that the gender classification is only binary in nature due to the data constraints and does not consider the non-binary genders. The study only considers the comments that are in the English language and may not be diverse enough in terms of language and culture. The capping and down sampling methods may result in representation bias. The study also uses automated annotation for sentiment analysis and may result in misclassifying the comments that contain sarcasm or implicit sentiment and comments that contain multiple languages.

Future work in the realm of analyzing sentiments in comments under STEM YouTube channels for male and female hosts holds significant potential for further insights and advancements. Conducting longitudinal studies to track changes in viewer sentiments over time could elucidate evolving trends and patterns within the STEM YouTube community. Understanding how sentiments fluctuate in response to changes in content, host demographics, or external events can provide valuable insights for content creators, platform administrators, and researchers.

## Data Availability

The raw data supporting the conclusions of this article will be made available by the authors, without undue reservation.
